# Comparing video and direct laryngoscopy for tracheal intubation in the general ward

**DOI:** 10.1186/s13613-018-0476-5

**Published:** 2019-01-30

**Authors:** Liu-Jia-Zi Shao, Fu-Shan Xue, Rui-Juan Guo, He Yang

**Affiliations:** Department of Anesthesiology, Beijing Friendship Hospital, Capital Medical University, NO. 95 Yong-An Road, Xi-Cheng District, Beijing, 100050 People’s Republic of China

Editor,

In a retrospective study by Baek et al. [1] comparing video laryngoscopy (VL) and direct laryngoscopy (DL) for intubation in the general ward, they showed that the use of VL was associated with a higher first-attempt success rate, but did not reduce intubation-related complications. The strengths of this study are a large sample and the use of consistent patients needing urgent intubation in the general ward by medical emergency team. Furthermore, the authors had applied right statistical methods including multivariable logistic regression analysis, propensity-score matching and subgroup analysis to determine associations of studied devices and intubation outcomes. Other than the limitations described in discussion, however, there are several issues in this study that need further discussion and clarification.

First, authors did not provide the positions of patient’s head during laryngoscopy and intubation. Because the use of DL to obtain the laryngeal visualization requires alignment of the oral, pharyngeal and laryngeal axes, a sniffing position is often recommended for intubation with DL. In contrast, the use of VL to visualize the larynx does not require alignment of three airway axes. Thus, intubation with VL has no specific requirement of patients’ head position [2]. We believe that ignorance of this factor would have biased the intubation outcomes in the favor of VL.

Second, to predict difficult airways, several factors including blood/vomitus/secretion in the airway, cervical immobilization, neck trauma/mass or vocal cord palsy, 3-3-2 rule, short neck, obesity, limited mouth opening, small mouth and large tongue, were assessed before intubation. It is usually considered that no single factor can accurately predict difficult airway as each factor individually has a rather low positive predictive value. If a patient has more predictors of difficult airway at the same time, however, the likelihood of difficult airway will increase [3]. For this reason, the National Emergency Airway Management Course has developed a LEMON score for identification of difficult airways in the emergency setting [4].

Third, main reasons for intubation in this study were respiratory failure and airway protection, but median intubation time with DL and VL was long up to 4 min. Other than first-attempt success rate, intubation time also is a concern for critical patients requiring urgent intubation, especially patients at risks of hypoxia and aspiration [5]. We are argued that this study would have provided more useful information about the choice of two intubation devices in the general ward, if a reasonable cutoff time had been included in the definition of first-attempt success rate.

Finally, experience and competency with VL and DL are critical for successful intubation. Because frequency and number of using two devices before study were not provided, the definitions that divided experienced and inexperienced intubators in this study could not accurately indicate the competency levels of intubators with VL and DL. Thus, it is difficult to determine whether a higher first-attempt success rate with VL is really attributable to a better performance. We are concerned that the results of this study may only map different learning curves of DL and VL for intubators, and do not measure the real efficiency of studied devices for urgent intubation in the general ward.

## Abbreviations

VL: video laryngoscopy
DL: direct laryngoscopy
